# Correlational study: illness representations and coping styles in caregivers for individuals with schizophrenia

**DOI:** 10.1186/1744-859X-12-27

**Published:** 2013-08-28

**Authors:** Shyhrete Rexhaj, Nataly Viens Python, Diane Morin, Charles Bonsack, Jérôme Favrod

**Affiliations:** 1 Community Psychiatry Service, Department of Psychiatry, University Hospital Centre of Vaud, Site de Cery, Prilly, 1008, Switzerland; 2 School of Nursing Sciences, La Source, University of Applied Sciences of Western Switzerland, Av. Vinet 30, Lausanne, 1004, Switzerland; 3 Institut Universitaire de Formation et de Recherche en Soins, University of Lausanne, Biopôle 2, Rue de la Corniche 10, Lausanne, 1010, Switzerland

**Keywords:** Caregivers, Representations of schizophrenia, Coping, Nursing care

## Abstract

**Background:**

Caring for individuals with schizophrenia can create distress for caregivers which can, in turn, have a harmful impact on patient progress. There could be a better understanding of the connections between caregivers’ representations of schizophrenia and coping styles. This study aims at exploring those connections.

**Methods:**

This correlational descriptive study was conducted with 92 caregivers of individuals suffering from schizophrenia. The participants completed three questionnaires translated and validated in French: (a) a socio-demographic questionnaire, (b) the Illness Perception Questionnaire for Schizophrenia and (c) the Family Coping Questionnaire.

**Results:**

Our results show that illness representations are slightly correlated with coping styles. More specifically, emotional representations are correlated to an emotion-focused coping style centred on coercion, avoidance and resignation.

**Conclusion:**

Our results are coherent with the Commonsense Model of Self-Regulation of Health and Illness and should enable to develop new interventions for caregivers.

## Background

### Problem statement

Between 30% and 91% of individuals with schizophrenia live in a family setting [1-3]. The decreased length of stay in hospital and restrictions on involuntary treatments mean that family-based caregivers provide an important support during periods of psychological instability. This support implies that caregivers rely on a variety of strategies to confront the consequences resulting from the psychological instability of the schizophrenia patient.

### Burden, distress, illness representations and coping strategies

The literature indicates that there are interactions between the different concepts of family burden, distress, illness representations, the expressed emotion (EE) and coping strategies. It has been demonstrated that caregivers’ representations of negative consequences of the illness for the patient are positively correlated with their objective burden, while representations of the negative consequences of schizophrenia for the caregiver are positively correlated to a subjective burden [4,5]. The caregivers’ subjective burden is also associated with negative emotional responses to the illness [5]. Also, caregivers having an elevated and hostile type of EE usually consider the patient responsible for the causes of illness [6,7]. Furthermore, in comparison with caregivers having a low EE, those having a high, critical-type EE tend to underestimate their possibilities of controlling problems themselves and perceive the illness as unlikely to be controlled by treatment as well as attribute more negative consequences of the illness for the patient and themselves [4].

Distress experienced by caregivers is also correlated with illness representations such as the following: (1) illness outbreaks are chronic, (2) the feeling that treatment does not help control the illness, (3) the perception that the patient can have greater personal control over the illness, (4) the perception that the illness brings about negative consequences for the patient and (5) that the illness brings about negative consequences for the caregiver and (6) representations that the illness elicits painful emotions such as anxiety and fear [4,5,8].

Coping strategies by caregivers of patients with schizophrenia have been explored by several authors who mainly based their observations using models or frameworks derived from Lazarus and Folkman’s theory of stress coping [9-12]. A review of the literature showed that there are strategies that are more or less efficient for confronting stress. More specifically for caregivers, strategies like coercion, avoidance and resignation are associated with suffering and patient relapse [13,14].

Birchwood and Cochrane [14] explored coping strategies used by caregivers of schizophrenia patients. Their results detail eight essential coping categories: coercion, avoidance, ignorance/acceptance, constructive, resignation, reassurance, disorganization and collusion. They also showed that coercion is the first predictor in patient relapses. As concerns specificities of different coping styles, Magliano et al. [15] conducted a study which explored coping strategies in relation to physical and somatic symptoms of caregivers as well as the association between these two variables. The Family Coping Questionnaire (FCQ) was used [10]. Their results indicated that emotion-focused coping (coercion, avoidance and resignation) is positively correlated with participant anxiety and depression [15]. In another study, Knudson and Coyle came up with a procedure which incited certain caregivers to modify their coping strategies [11]. In their qualitative study, the caregivers of individuals with schizophrenia were invited to describe their coping strategy. At the onset of the illness, the caregivers tended to use a problem-focused coping strategy. However, if the symptoms became persistent, they progressively opted for emotion-focused strategies, which enabled them to attain a position of acceptance and, ultimately, of well-being. Nevertheless, the conceptualization of emotion-focused coping done according to factor analysis in the study of Magliano et al. only included strategies such as resignation, avoidance and coercion [10]. Acceptance of the illness has seldom been studied and could be a potential functional human response in some instances.

Magliano et al. reported that the high level of burden is associated with a reduction in social interests, a reduction of social support network as well as a resignation and an avoidance of contact with the family member suffering from schizophrenia. It also appears that in the absence of any specific intervention with these caregivers, this result can remain unchanged for as long as a year [15]. Specific to the subjective burden, it appears that emotional and cognitive reactions are associated with the use of coping strategies that are specifically emotional, such as avoidance and isolation [16,17]. These strategies have been identified as being linked to an increase in caregiver distress [18]. Increased distress can result in a deterioration of the family atmosphere through elevated EE levels [12]. These elements lead to a further increase in burden [19].

### Theoretical framework

In terms of theory, in the 1960s, Leventhal and his colleagues developed the Commonsense Model of Self-Regulation of Health and Illness (SRM) [20]. The results from their initial research indicated that modifying the personal representations of health and the development of an action plan were the two determining factors for the creation of health-promoting actions. Their conclusions led to the development of a series of other studies meant to define the different characteristics of health and illness representations. According to the model, representations are cognitive and emotional constructions of a health problem. The SRM includes eight concepts or dimensions: (1) internal and external stimuli, (2) treatment system, (3) representation of the illness and the treatment, (4) coping procedures, (5) evaluation of the cognitive treatment of the information, (6) emotional representation, (7) coping responses and (8) evaluation of the emotional treatment of the information.

Coping involves procedures which enable an individual to collect information and control the problem, as well as different responses such as distraction or relaxation. Coping can be described as having two primary functions. The first is centred on problem solving and the second on the immediate regulation of the emotion elicited by the problem. According to the SRM, the choice of a coping strategy used by caregivers is associated with the kind of representation of the illness that the caregiver has developed. In this way, the caregiver’s explicative model seems to have an influence on their choice of coping strategy. This choice can then, in turn, lighten or increase caregiver burden [17]. Effectively, negative representations of the illness can lead to the use of unsuitable coping strategies [21,22].

### Goal of the study

The goal of this study was to explore the associations between illness representations and three forms of coping styles—(1) problem-focused coping, (2) emotion-focused coping and (3) social support-focused coping—for caregivers of individuals with schizophrenia.

## Methods

### Design and recruitment

This correlational descriptive study was conducted with 92 caregivers of individuals with schizophrenia. Participants were members of French-speaking social support organizations, were recruited using a convenience sampling strategy and met the following criteria: (1) being 18 years or older, (2) living in Switzerland or France, (3) being able to speak French, (4) acting as carer for a family member or close friend who suffers from schizophrenia and (5) having had at least a 1-h contact with this person in the last month. As the first step of the recruiting strategy, social support organizations were met at the time of meeting with members in order to provide a complete presentation of the study. Then, the members who were present received an envelope containing an informed consent sheet, an information letter and the necessary questionnaires, as well as a pre-addressed and pre-stamped envelope for the return of all completed documents.

A second recruiting strategy was conducted using an electronic survey. The presidents of the social support organizations sent to all members of their respective groups the survey link leading to a dedicated website that included the same documentation as the paper version.

### Instruments

Participants filled out three self-administered forms: (a) a socio-demographic questionnaire, (b) the *Illness Perception Questionnaire for Schizophrenia*: *Relatives’ version* and (c) the *Family Coping Questionnaire.* Authorization to translate these questionnaires into French using independent backward translation was granted by the authors.

#### The socio-demographic questionnaire

According to the literature, the most significant socio-demographic variables which influence illness representations and coping strategies are the following: (1) caregiver gender [23], (2) caregiver age, (3) caregiver education level [13], (4) the length of contact with the patient [19,24], (5) professional and social support [25,26] such as involvement in a Profamille program [27], (6) the nature of their connection with the patient [28], (7) the length of the illness [29], (8) whether the caregiver lives with the patient or not [30], (9) age of the patient, (10) patient gender and (11) the patient’s professional support. With the further goal of comparing our results with other studies already reported in the literature, we collected data on the number of people living in a household and the civil status of the caregivers.

#### The Illness Perception Questionnaire for Schizophrenia: Relatives’ version

Illness representations were measured using a self-administered questionnaire entitled ‘Illness Perception Questionnaire for Schizophrenia: Relatives’ version (IPQS: Relatives)’ [5]. It involves 13 sub-scales (150 items): identity, timeline (acute/chronic), timeline (cyclic), negative consequences for the individual, negative consequences for the caregiver, personal control (feeling of powerlessness—patient), personal control (feeling of powerlessness—caregiver), personal blame (patient responsibility), personal blame (caregiver responsibility), therapeutic control, mental health problem coherence, emotional representations and causes. For the current study, the identity and the causes of the illness were not considered. The responses provided by the caregivers regarding their perceptions of the illness were measured using a 5-level Likert scale: 0 = do not agree at all, 1 = do not agree, 2 = more or less agree = 3 = agree and 4 = completely agree. Results show a dimensional reliability situated between α = 0.63 and α = 0.83 [5].

#### The Family Coping Questionnaire

Coping styles were measured using a self-administered questionnaire entitled ‘Family Coping Questionnaire (FCQ)’. The version of the FCQ, used in this study, included 27 items measuring seven dimensions (information gathering, positive communication, social involvement, coercion, avoidance, resignation, the patient’s social involvement). A factor analysis enabled the authors to identify three main factors. Looking at these three factors and at the conceptual definition of coping, it is clear that the seven dimensions can be regrouped into three coping modes: (1) problem-focused coping (the patient’s social involvement, positive communication and information gathering are positively correlated with each other and negatively with avoidance, (2) emotion-focused coping (coercion, avoidance and resignation are positively correlated) and (3) social support-focused coping (social involvement is associated with avoidance) [10]. Our study catalogued these strategies. Caregiver responses to different situations were measured using a 5-level Likert scale: 1 = never, 2 = rarely, 3 = sometimes, 4 = very often and 5 = not applicable. Its validity was demonstrated during the BIOMED 1 study, conducted in five European countries [15]. Results showed a dimensional reliability between *α* = 0.68 and *α* = 0.83 [13].

### Data analysis

Data were treated using the computing program ‘IBM SPSS Statistics® version 20’. Descriptive statistics were used to describe socio-demographic characteristics. To test associations between illness representations and coping styles, bivariate correlational analyses were used. The *p* value threshold used was set to <0.05.

### Ethical considerations

All participants were required to sign an informed consent form for the paper version of the survey or confirm their consent in order to access the electronic version of the survey. The research protocol received full authorization by the Canton of Vaud’s Ethics Committee for human-based research.

## Results and discussion

While Table 1 presents the socio-demographic characteristics of participants, Table 2 presents the main characteristics of the individuals with schizophrenia cared for. As showed, the final sample involved 92 middle-aged individuals (mean age = 56 years, standard deviation (SD) = 12.6) including 68% of women. Most participants were married or lived in a household as a married couple (70.3%). The majority of respondents were either mothers or fathers (66.3%) of the individual with schizophrenia. All participants had completed some level of education; vocational school, apprenticeship and university-level education were the most often cited. Most participants reported that they lived with one or several other individuals. Most of them (62%) participated to the Profamille program, a psycho-educational program for family members and friends of individuals with schizophrenia [31]. Among the 92 participants, 37 (40.2%) reported that they lived with the schizophrenia patient.

**Table 1:** Socio-demographic characteristics of participants

**Characteristics**	**Value**
Age, *N* = 92 (mean score (SD))	56.43 (12.61)
Number of people in household, *N* = 92 (mean score (SD))	2.88 (1.49)
Sex (*N* (%))	92 (100)
Female	63 (68.5)
Male	29 (31.5)
Status (*N* (%))	91 (100)
Single	9 (9.9)
Married	64 (70.3)
Widowed	8 (8.8)
Separated/divorced	10 (11.0)
Relationship type (*N* (%))	92 (100)
Mother/father	61 (66.3)
Daughter/son	10 (10.9)
Sister/brother	8 (8.7)
Other parent	4 (4.3)
Wife/husband	6 (6.5)
Friend	0
Neighbour	0
Colleague/school friend	0
Others	3 (2.8)
Completed education level (*N* (%))	92 (100)
Compulsory education	4 (4.3)
Apprenticeship	17 (18.5)
Maturity, high school	18 (19.6)
School profession	31 (34.8)
University	21 (22.8)
Number of people in household (*N* (%))	91 (100)
1	8 (8.8)
2	36 (39.6)
3	22 (24.2)
4	7 (7.7)
5	10 (11.0)
6	4 (4.4)
7	2 (2.2)
Profamille psycho-educational program (*N* (%))	92 (100)
Yes	57 (62.0)
No	20 (21.7)
Under way	15 (16.3)

**Table 2:** Characteristics of individuals with schizophrenia

**Characteristics**	**Value**
Duration of illness, years (mean score (SD))	15.05 (9.67)
Age (mean score (SD))	33.77 (11.56)
Number of therapist (mean score (SD))	1.97 (1.15)
Sex (*N* (%))	92 (100)
Female	21 (22.8)
Male	71 (77.2)
Living under the same roof (*N* (%))	92 (100)
Yes	37 (40.2)
No	55 (59.8)
Community services used (more than one service) (*N* (%))	92 (100)
Psychiatrist	81 (88.0)
General practitioner	32 (34.8)
Nurse	31 (33.7)
Psychologist	12 (13.0)
Social worker	19 (20.7)
Occupational therapist	6 (6.5)
Sheltered workshop	31 (33.7)
Day centre	16 (17.4)

As concerns the individual with schizophrenia, results show that the duration of the illness is long (mean = 15.1 years, SD = 9.7). The mean age of the individual with schizophrenia taken care for by the participants of this study was 34 years and 77.2% were male. Individuals with schizophrenia call upon community services in a variety of ways. In 88% of all cases, a psychiatrist was involved in patient follow-up. In 34.8% of all cases, a general medical practitioner was involved in patient follow-up. In our sample, 33.7% of the schizophrenia patients received nursing care. Also, a significant number (33.7%) of the individuals with schizophrenia in our sample were involved in sheltered workshops. Other community services were also used at more than 10% (social worker, 20.7%; psychologist, 13%; day centre, 17.4%).

Results presented in Table 3 show that caregivers have a great conception of the illness as being chronic and as having recurring symptoms per cycle. They have a strong perception that the illness brings about negative consequences for the individual with schizophrenia. While at the same time, they perceive fewer negative consequences for themselves caused by the patient’s illness. They have a feeling of control over the illness and that the patient can also have control. They do not think that the patient is responsible for his or her illness. They do not see themselves as responsible for the appearance of the illness. Treatment is perceived as helpful for better controlling the illness. For these caregivers, the illness has meaning and coherence. They more or less agree that the illness brings about negative emotions like sorrow or anxiety, measured here under the category of emotional representations. Participants in this study mostly used problem-focused and social support-focused coping styles. Emotion-focused coping was used less compared to the two other styles (median = 15, min to max = 7.00–28.00).

**Table 3:** Mean and median scores for IPQS-relatives sub-scales and coping styles

	**Number of items**	**Mean score (SD)**	**Median score (min to max)**
IPQS-relatives sub-scale			
Timeline (acute/chronic)	6	3.27 (0.56)	3.33 (1.83–4.00)
Timeline (cyclical)	4	3.01 (0.62)	3.00 (1.00–4.00)
Negative consequences (caregiver)	9	1.46 (0.61)	1.44 (0.11–3.22)
Negative consequences (patient)	11	2.90 (0.54)	3.00 (1.36–4.00)
Personal control (caregiver helplessness)	4	2.34 (0.82)	2.25 (0.25–4.00)
Personal control (patient helplessness)	4	2.57 (0.78)	2.50 (0.25–4.00)
Personal blame (caregiver responsibility)	3	0.94 (0.70)	1.00 (0.00–2.67)
Personal blame (patient responsibility)	3	0.76 (0.75)	0.67 (0.00–3.33)
Treatment control	5	2.50 (0.70)	2.60 (0.20–4.00)
Coherence	5	1.05 (0.72)	1.00 (0.00–2.60)
Emotional representation	9	1.90 (0.72)	1.89 (0.22–3.78)
Coping styles			
Problem-focused coping	11	30.71 (7.28)	32.00 (7.00–43.00)
Emotion-focused coping	10	15.60 (4.40)	15.00 (7.00–28.00)
Social support-focused coping	8	22.33 (3.77)	23.00 (8.00–28.00)

Correlations between illness representations and coping styles are presented in Table 4. It can be seen that all the statistically significant correlations are somewhat low ranging from *r* = 0.23 to *r* = 0.41. The statistically significant correlations are the following:

1. Representations that the illness brings about negative consequences for the caregiver have a moderate positive correlation with problem-focused coping (*r* = 0.31, *p* = 0.006) and emotion-focused coping (*r* = 0.35, *p* = 0.002). There was also a slight negative correlation between these representations and social support-focused coping (*r* = −0.29, *p* = 0.012).

2. Representations that the illness brings about negative consequences for the patient have a slight positive correlation with emotion-focused coping (*r* = 0.23, *p* = 0.037).

3. The presence of a feeling of control by the caregiver is moderately positively correlated with problem-focused coping (*r* = 0.31, *p* = 0.006).

4. Representations that the caregiver might be responsible for the onset of the illness have a moderate positive correlation with emotion-focused coping (*r* = 0.34, *p* = 0.001).

5. Representations that the patient is responsible for the onset of the illness have a slight positive correlation with emotion-focused coping (*r* = 0.25, *p* = 0.020).

6. Representations that treatment helps to control the illness have a slight positive correlation with problem-focused coping (*r* = 0.23, *p* = 0.040).

7. A lack of meaning attributed to the illness has a moderate positive correlation with emotion-focused coping (*r* = 0.31, *p* = 0.005).

8. Representations that the illness brings about negative emotions have a moderate positive correlation with emotion-focused coping (*r* = 0.41, *p* = 0.000) and a moderate negative correlation with social support-focused coping (*r* = −0.39, *p* = 0.000).

**Table 4:** Correlations between illness representations and coping styles

**Variables**		**Problem-focused coping**	**Emotion-focused coping**	**Social support-focused coping**
Negative consequences (caregiver)	*r*	0.31	0.35	−0.28
	*p*	0.006*	0.002*	0.012*
Negative consequences (patient)	*r*	0.06	0.23	−0.03
	*p*	0.616	0.037*	0.818
Personal control (caregiver helplessness)	*r*	0.31	−0.10	−0.02
	*p*	0.006*	0.380	0.797
Personal blame (caregiver responsibility)	*r*	0.11	0.34	−0.01
	*p*	0.316	0.001*	0.953
Personal blame (patient responsibility)	*r*	−0.15	0.25	−0.10
	*p*	0.155	0.020*	0.361
Treatment control	*r*	0.23	−0.04	0.09
	*p*	0.040*	0.705	0.407
Coherence	*r*	−0.09	0.31	−0.16
	*p*	0.432	0.005*	0.140
Emotional representation	*r*	0.13	0.41	−0.39
	*p*	0.262	0.000*	0.000*

*r* Pearson correlation test, *p* probability test. *p < 0.05, significant probability test.

It is timely to note that this study is the first known to use French-validated tools to measure correlations between representations of schizophrenia and coping styles within a caregiver sample. For most variables, our results align with several other studies in this field [8,9,13,15,32]. Nevertheless, the caregivers in this study were less likely to have the perception that the patient was at fault or that they themselves were responsible for the onset of the illness than compared to the results published by Lobban and collaborators [5]. Our results also show that emotion-focused coping is moderately correlated with (1) negative consequences for the caregivers, (2) the feeling of being at fault, (3) the feeling that the mental health problem is not coherent and (4) the overall score of the emotional representation scale. Problem-focused coping is itself moderately correlated with (1) the perception of negative consequences for caregivers and (2) the feeling of control. Social support-focused coping has a moderate negative correlation with the overall scores of the emotional representation scale. The other correlations are non-significant or less than 0.30 with a slight scaling effect [33].

The SRM predicts that illness representations influence coping procedures and that emotional representations influence coping responses. These different factors also influence each other. More specifically, when a health threat occurs, it is handled by the *processing system*, the second concept of SRM. This system consists of two types of threat management: (1) the representations of illness and treatment and (2) the emotional representations. Both types can influence each other. Each type of threat management includes three steps to process information: representation (illness and treatment), coping procedures and evaluation. Regarding the first type of threat management, the first stage of data processing is the development of a representation of the disease and the treatment. This concept determines the goals and coping procedures to achieve them [34]. Coping procedures are the second stage of information processing. They are a wide range of cognitive and behavioural measures undertaken in response to the cognitive representation, such as problem-focused coping [22,34]. The third step of information processing is the evaluation of the cognitive treatment of information. Regarding the second type of threat management, the first step of information processing is the formation of an emotional representation [22]. Based on this emotional representation, goals are set and coping responses are determined to achieve them. Coping responses are the second step of information processing. They are a wide range of strategies focused on managing emotions that are applied in response to the emotional representation. The last step of information processing for this second type is the evaluation of the emotional treatment of the information. The results of the evaluation step produce a feedback at the preceding steps*.*

Our results are quite coherent with this predictive model, especially for emotion-focused coping and the score of emotional representations, consequences for the caregiver and the feeling of being at fault. Consequences for the patient and the feeling of control are associated with problem-focused coping. The fact that the consequences for the caregiver are associated as much with problem-focused coping as with emotion-focused coping underscores the presence of interactions between the emotional and cognitive variables.

A study similar to ours [8] did not establish a positive correlation between the representations of the illness that bring about negative consequences for the caregiver and problem-focused coping. It is important to note that these authors did not conceptualize coping as we did, and therefore, comparing our results is a limited exercise. However, the abovementioned correlation leads one to think that the Profamille psycho-educational program followed by a significant portion of our participants had an impact on this coping style without actually modifying the representations of the illness as conceived by the participants.

Our sample pool was particular in that it was composed of individuals belonging to family-type assistance programs and that most of the participants had followed some kind of psycho-educational program. On average, our participants had been caring for their patient for 15 years. This could explain the weak level of reliance on emotion-focused coping. Nevertheless, the correlations observed suggest that the SRM model is still valid over time.

In terms of intervention, the psycho-educational programs available for the caregivers tend to focus on knowledge acquisition of the illness and treatment and positive communication skills training to reduce stress within the family. Our data suggest that it would be of interest to take better into account the emotional representations of the illness. In everyday practice, first-line health care professionals have to care for caregivers’ painful emotions such as anger, guilt, sadness and fear. However, the required skills to help caregivers to better manage their painful emotions are difficult to define precisely. The development and evaluation of the best professional strategies to help caregivers to better manage their painful emotions appear to be an interesting line of research for the future. Refining the best professionals’ skills to help caregivers to deal with negative emotions may influence more directly emotional representations and coping skills in caregivers.

Given the scarcity of studies to compare to, further studies could be useful to better identify coping styles that improve emotional regulation without negative consequences for the patient, like acceptance or changing value systems. In this way, a holistic understanding of care, as suggested by several authors [35,36], could be further promoted. Thus, interventions could be focused on the development of alternatives to less effective or even painful strategies like resignation, coercion and avoidance. Furthermore, the tools used to measure emotion-focused coping tend to highlight more negative emotional coping methods like coercion, avoidance or resignation. It is important to develop a measuring instrument that also takes into account the positive strategies focused on emotion.

### Limitations of the study

This was the first study using the IPQS: Relatives and the FCQ in French. Participant recruitment took place within the context of social support organizations according to a convenience sampling method. Therefore, our results could be cautiously generalized to other programs having a similar context to ours, in terms of culture and health care systems.

## Conclusion

In the empirical literature, as well as in the Commonsense Model of Self-Regulation of Health and Illness, problem-focused and social support-focused coping styles are recognized as efficient for decreasing caregiver loads and increasing a patient’s chances of better coping with schizophrenia. The results of this study also show that illness representations influence the choice of coping styles. The coping style approach is useful to develop further targeted and feasible interventions. Further studies in this field should focus on the evaluation of interventions based on illness representations in order to prevent the use of unsuitable coping styles. Also, it will be necessary to identify this phenomenon in a caregiver population of individuals who are not involved into any mutual support group in order to adapt appropriate care and support procedures.

## Abbreviations

EE: Expressed emotion; FCQ: Family coping questionnaire; IPQS: Relatives, illness perception questionnaire for schizophrenia: relatives’ version; SD: Standard deviation; SRM: Commonsense model of self-regulation of health and illness.

## Competing interests

The authors declare that they have no competing interests.

## Authors’ contributions

SR, DM and JF contributed to the conception and design of the study. SR contributed to the acquisition of data. SR and JF performed the statistical analysis and drafted the first manuscript. NVP and CB critically reviewed and revised the manuscript. All authors read and approved the final manuscript.
